# Functional Analysis of the Rhoptry Kinome during Chronic *Toxoplasma gondii* Infection

**DOI:** 10.1128/mBio.00842-16

**Published:** 2016-06-14

**Authors:** Laura J. Knoll

**Affiliations:** Department of Medical Microbiology and Immunology, University of Wisconsin—Madison, Madison, Wisconsin, USA

## Abstract

*Toxoplasma gondii* is one of the most common parasitic infections of humans worldwide. Once exposed, humans remain infected with *T. gondii* for life, and there are no therapeutics capable of eliminating a chronic infection. In the search for novel drug targets, *T. gondii* is known to contain several unique secretory organelles, one of which is called the rhoptries. Rhoptry organelles contain and secrete numerous proteins with kinase domains, but the roles of most of these kinases during infection remain unknown. In a recent mBio article, B. A. Fox et al. [mBio 7(3):e00193-16, 2016, http://dx.doi.org/10.1128/mBio.00193-16] performed a tour de force deletion analysis of 31 rhoptry kinases and examined their roles in the development of chronic infection. While rhoptry kinase deletion strains that displayed an acute infection defect also showed a reduction in chronic infection cyst burden, two rhoptry kinase deletion strains had decreased cyst burden without any change in acute virulence. These results indicate the necessity of the rhoptry kinases for the establishment and perhaps maintenance of chronic infection. They also highlight the potential of these kinases as drug targets to clear chronic infection or as candidates to generate a nonpersisting vaccine.

## COMMENTARY

The parasite *Toxoplasma gondii* is the most common parasitic infection worldwide because it can infect any warm-blooded animal and persists throughout the host’s life span. Because of that lifelong chronic infection, antibody titers against *T. gondii* remain high and have been used to measure its seroprevalence. There is a high seroprevalence of *T. gondii* in wildlife throughout the world in terrestrial as well as marine environments (1). While the seroprevalence in humans has decreased over the last 30 years, it still remains high in many areas of the world, and it is likely that one-third of the world’s human population harbors a chronic *T. gondii* infection (2). *T. gondii* was discovered more than 100 years ago and is known to cause congenital infections in developing fetuses. Toxoplasmic encephalitis most often occurs in immunocompromised patients when the chronic cyst stage reactivates as cellular immune surveillance is lost (3); *T. gondii* was notorious during the AIDS pandemic as a major cause of encephalitis. Having therapeutics that could eliminate bradyzoite cysts or prevent their reactivation would be highly valuable treatment for patients before their immune response becomes too limited.

*T. gondii* has a complex life cycle that contains both sexual and asexual phases. The sexual cycle of *T. gondii* is restricted to the feline intestine where several unique stages develop before differentiation into macrogametes and microgametes (4). The macro- and microgametes fuse to produce diploid oocysts, which develop thick, impermeable walls and are shed in the feces. In ambient air and at ambient temperature, oocysts mature by undergoing mitosis and meiosis to contain eight haploid sporozoites encased within the oocyst wall. The asexual cycle of *T. gondii* is less complex and can occur within any warm-blooded animal. The asexual cycle has two developmental stages: a rapidly replicating form called a tachyzoite and a slow-growing form called a bradyzoite that is the hallmark of chronic life-long infection. Tachyzoites do not form a cyst wall around their parasitophorous vacuole (PV), whereas bradyzoites do, causing bradyzoite parasites to be resistant to pepsin and acid. Because the bradyzoite tissue cyst is infectious, the sexual cycle of *T. gondii* is not mandatory for transmission, and the asexual cycle can propagate solely through carnivorism. In humans, ingestion of bradyzoite cysts in undercooked meat is considered the primary route of exposure (5, 6), but antibodies specific to oocyst stages have been detected, suggesting that oocyst contamination of food and water is also a source of infection (5, 7).

*T. gondii* is a member of the apicomplexan phylum that contains some of the most devastating human parasites, including *Plasmodium* and *Cryptosporidium* species. In the search for novel drug targets, proteins in the unique secretory organelles of apicomplexan parasites have been highlighted as having excellent potential. The best characterized of these secretory organelles are the micronemes, rhoptries, and dense granules, which are sequentially secreted during attachment, invasion, and establishment of the PV. Each of these organelles contains many proteins, some of which appear to be functionally redundant. For example, the rhoptries of *T. gondii* are predicted to contain approximately 50 kinases and pseudokinases (8, 9). Given the complexity of apicomplexan life cycles, it is likely that these rhoptry paralogs perform their functions during different life stages. Certain kinases may not play a role during the tachyzoite stage but may instead be necessary to establish or maintain chronic infection or be essential in the sexual stages within the cat. Investigating the roles of these rhoptry kinases during different life cycle stages opens a window to determine novel drug targets that are active against alternative stages, such as bradyzoite cysts.

It is against this background that the work by Fox et al. (10) stands out as highly significant. They performed a comprehensive deletion analysis of the rhoptry kinome and specially determined which ones are needed for chronic infection establishment and likely maintenance. They deleted 32 identified members of the rhoptry kinase gene family in a type II strain of *T. gondii*. Some of these rhoptry kinases had already been deleted and characterized in type I strains of *T. gondii*, but type I strains are so virulent that all infected mice die during acute infection and do not progress to the chronic cyst stage. Fox et al. deleted 32 rhoptry kinases in a type II strain so that they could examine acute and chronic infection defects. Type II strains are also responsible for the majority of human infections in Europe and North America (11, 12); however, *T. gondii* strains from humans in South America have more genetic diversity (13–15).

While most of the deletion strains were not significantly reduced or were only modestly reduced in their ability to produce bradyzoite cysts, five deletion strains had drastic reductions in cyst burden and one deletion strain had a significant increase in the number of cysts. None of the five deletion strains with severe decreases in cyst burdens had any defects in bradyzoite cyst development when examined in a tissue culture model, indicating that these mutants had intact mechanisms to form cysts but were inhibited specifically during animal infection. The three deletion mutants with the most severe cyst reductions, Δ*ROP5*, Δ*ROP17*, and Δ*ROP18* strains, have previously been shown to have acute infection defects in type I strains. ROP5 and ROP18 had been determined to be key virulence factors in multiple studies (16–21), and ROP17 associates with ROP5 in a macromolecular complex (22). It is likely then that the severe reduction in cyst burden seen for Δ*ROP5* and Δ*ROP18* mutants is linked to their important role during acute infection and may not be specific to the chronic stage. The Δ*ROP17* strain had increased virulence during acute infection compared to Δ*ROP5* and Δ*ROP18* strains. This difference in acute virulence along with the almost 100% reduction in cyst burden for the Δ*ROP17* mutant indicates that ROP17 plays important roles during both acute and chronic infection.

ROP5 and ROP18 have well-defined roles during acute infection in resisting innate immune responses stimulated by gamma interferon. ROP18 is located on the cytoplasmic side of the PV membrane (PVM), where it phosphorylates host immunity-related GTPases (IRGs) and prevents destruction of the PV (22–25). The mouse IRG family contains 23 members, whereas the human genome contains only one full-length ortholog without interferon-inducible elements in its promoter (26). As mice are the primary reservoir for *T. gondii*, the parasite has evolved a complex system to combat IRG-mediated destruction in its major host. ROP5 is a pseudokinase, but its association with ROP18 increases kinase activity (27). Type I strains of *T. gondii* are highly resistant to host IRG killing, whereas type II strains are degraded by host IRGs because ROP5 from type II strains are not effective at inhibiting destruction by host IRGs. Fox et al. (10) show that survival of Δ*ROP5* and Δ*ROP18* mutants are further reduced with increased IRG association around the PVM and that the additional damage cannot be complemented by a kinase-dead or non-PVM-associating ROP18. ROP17 did not appear to contribute to this IRG resistance phenotype in type II strains; however, the Δ*ROP17* mutant did display small-plaque and early egress phenotypes that were not connected with parasite growth rate. These phenotypes may be contributing to both the acute and chronic infection virulence defects seen in the Δ*ROP17* mutant.

Two deletion mutants, Δ*ROP35* and Δ*ROP38*/*29*/*19* (*ROP38*, *ROP29*, and *ROP19* deleted), had dramatic reductions in cyst burdens but did not have changes in virulence during acute infection. Rhoptry kinases 38, 29, 19A, and 19B are located sequentially on chromosome VI, so the authors were able to remove all four genes with a single deletion. This Δ*ROP38*/*29*/*19* strain as well as the Δ*ROP35* strain has the same virulence as the parental strain when mice were challenged with a dose just twofold above the 50% lethal dose. These Δ*ROP35* and Δ*ROP38*/*29*/*19* mutants were decreased in cyst burden by 75 to 80% compared to the parental strain. These studies set the stage for further analysis of these two mutants, including the long-term stability of the cysts during chronic infection, if those cysts are infectious to the next host, and if they can start the sexual cycle in cats.

As mentioned previously, toxoplasmic encephalitis most often occurs in immunocompromised patients when the bradyzoite cysts reactivate (3), but it can also occur when *T. gondii* is acquired by the ingestion of undercooked meat or oocysts from a cat. Animal vaccines that prevent infectious cyst formation in livestock or oocyst production in cats would greatly reduce transmission of *T. gondii* to humans. Investigations such as these by Fox et al. (10) lay the groundwork for development of novel therapeutics active against bradyzoite cysts and future vaccine strains that reduce transmission to humans.
